# Integrating Health Belief Model and Human Factors Engineering to Prevent Musculoskeletal Injuries Among Operating Room Nurses: A Quality Improvement Prospective Pilot Study

**DOI:** 10.3390/healthcare14142046

**Published:** 2026-07-08

**Authors:** Allen Herng Shouh Hsu, Chun Hung Chen, Jui-Ting Wei, Man Ju Wei, Po Chun Lin, Chung Fang Li, Pei-Shan Lee

**Affiliations:** 1 Department of Orthopaedic Surgery, Kaohsiung Chang Gung Memorial Hospital, Kaohsiung 833, Taiwan; alking0061@cgmh.org.tw (A.H.S.H.); wei0728@cgmh.org.tw (J.-T.W.); u301117@cgmh.org.tw (P.C.L.); 2 Department of Nursing, Kaohsiung Chang Gung Memorial Hospital, Kaohsiung 833, Taiwan; night@cgmh.org.tw (C.H.C.); fun1115@cgmh.org.tw (C.F.L.); 3 Department of Medical Education, Kaohsiung Chang Gung Memorial Hospital, Kaohsiung 833, Taiwan; weiwei0617@cgmh.org.tw

**Keywords:** operating room nurses, work-related musculoskeletal disorders, human factors engineering, Health Belief Model, self-efficacy, ergonomics, occupational safety

## Abstract

**Highlights:**

**What are the main findings?**

- A multifaceted ergonomic intervention was associated with improved musculoskeletal injury prevention awareness among operating room nurses.
- Fewer non-ergonomic actions were observed following implementation of the intervention, with improvements maintained through 6-month follow-up.

**What are the implications of the main findings?**

- Integrating behavioral education with human factors engineering may provide a promising strategy to reduce WMSDs in high-risk clinical environments.
- This approach may serve as a scalable model for improving occupational safety and workforce sustainability in healthcare environments.

**Abstract:**

**Background**: Operating room (OR) nurses are at high risk for work-related musculoskeletal disorders (WMSDs) due to physically demanding tasks and the use of heavy lead aprons. **Objectives**: This study evaluated the impact of a multidimensional ergonomic intervention on injury prevention awareness, compliance, and health outcomes among OR staff. **Methods**: A prospective quality improvement pilot study was conducted with 33 OR nurses. The intervention, based on the Health Belief Model and Human Factors Engineering, integrated ergonomic education with environmental modifications. Outcomes were assessed via questionnaires, field audits, and sick leave records at baseline, post-intervention, and 6-month follow-up. **Results**: Awareness scores improved from 68.7% to 98.8% (*p* < 0.001). Observational audits demonstrated a reduction in non-ergonomic actions from 2227 to 440 (mean deviations per nurse: 111.4 to 22.0, *p* < 0.001), with improvements sustained at 6 months. Annual sick leave cases decreased from three to one. **Conclusions**: A multifaceted real-world pilot intervention combining education with system-level engineering was associated with enhancing ergonomic compliance and reducing unsafe behaviors. This approach fosters the self-efficacy necessary for sustainable WMSD risk mitigation in high-intensity clinical settings.

## 1. Introduction

Work-related Musculoskeletal Disorders (WMSDs) constitute a major occupational health burden worldwide [1]. Epidemiological evidence from Europe indicates a substantial prevalence of musculoskeletal pain involving the back, neck, and upper limbs, affecting a large proportion of the working population [2]. A similar pattern has been reported in Taiwan, where a recent study among hospital nurses demonstrated a high prevalence of musculoskeletal symptoms, particularly involving the shoulder, lower back, and neck [3].

While ergonomic interventions have been shown to reduce the risk of work-related musculoskeletal disorders among healthcare workers, many existing studies have focused on isolated educational initiatives or workplace ergonomic modifications. Although multifaceted intervention strategies have also been explored, there remains limited evidence regarding the integration of behavioral change theory with Human Factors Engineering in highly specialized operating room environments. Furthermore, few studies have simultaneously evaluated knowledge acquisition, observed ergonomic behaviors, and occupational health outcomes following a multifaceted ergonomic intervention. Therefore, the present study was designed to evaluate a multifaceted intervention integrating the Health Belief Model with Human Factors Engineering among orthopedic operating room nurses, a workforce exposed to unique ergonomic demands.

Our institution previously conducted an investigation on an orthopedic operating unit, indicating that 74.8% of Operating Room (OR) nurses experienced musculoskeletal symptoms within a one-year period. Notably, the shoulder emerged as the most affected area, impacting 77.6% of the respondents. Key contributing factors include unfavorable postures, excessive force exertion, repetitive movements, and prolonged physical stress. While rarely life-threatening, WMSDs require prolonged recovery periods that lead to a decline in functional capacity and quality of life. Furthermore, the associated rise in medical costs and lost productivity imposes a significant burden on society [4].

The inherent physical demands of operating room duties place nurses at increased risk of WMSDs. These include prolonged standing, routine use of heavy protective lead aprons, and transporting surgical trays or equipment weighing several hundred kilograms [5]. Nurses often adopt poor ergonomic postures to maintain operational efficiency, reflecting a critical lack of knowledge regarding body mechanics among perioperative staff [6].

A preliminary questionnaire-based investigation demonstrated that self-efficacy and nursing credentials (tenure) were the strongest predictors of musculoskeletal injury prevention behaviors, as identified through multivariable logistic regression analysis (Table 1). However, empirical evidence regarding effective ergonomic interventions for OR nurses in the local clinical context remains limited. Addressing this gap requires a dual approach that targets both individual behavioral determinants and the physical work environment. Human Factors Engineering (HFE) provides a robust framework for optimizing the interface between nursing staff and their work system, ensuring that safety is designed into the clinical workflow [7]. Building upon HFE principles and the theoretical framework of the Health Belief Model [8], a prospective ergonomic and educational intervention was conducted to translate these behavioral insights into a multifaceted ergonomic prevention strategy. This approach proposes that a combination of cognitive reinforcement and environmental modification is essential for fostering self-efficacy and sustaining long-term behavioral change. Specifically, a structured program was designed to enhance ergonomic awareness, improve work safety, and reduce the occurrence of musculoskeletal injuries. The intervention aimed to strengthen preventive behaviors, decrease nursing workforce attrition, and promote the physical and mental well-being of OR nurses.

## 2. Materials and Methods

### 2.1. Participants

The study subjects belong to a surgical unit comprising seven orthopedic operating rooms (ORs) staffed by 35 nurses. The entire orthopedic nursing staff participated in the pilot study. Regarding professional tenure, 2 nurses (5.7%) had less than 5 years, 10 (28.6%) had between 5 and 10 years, and the majority (23, 65.7%) had more than 10 years of experience. Each OR is staffed by one circulating nurse and one scrub nurse, responsible for assisting surgeons and managing surgical instruments. In 2023, the unit performed a total of 6980 orthopedic surgeries, averaging 580 cases per month. On average, each nurse assists with four to five surgeries per day. Of the 35 nurses in the unit, 33 completed the baseline (pre-test) musculoskeletal injury prevention awareness questionnaire and were included in the pre- and post-test analysis. Two questionnaires were considered invalid due to incompletion and failure to remain anonymous.

### 2.2. Study Design

This study utilized a real-world quality improvement and exploratory intervention design. Following an initial questionnaire survey of operating room nurses, and a preliminary ergonomic assessment, a structured ergonomic and educational intervention program was developed tailored to identified behavioral predictors (Table 1). The intervention was implemented within the same cohort of nurses who participated in the baseline survey, and effectiveness was evaluated by comparing pre- and post-intervention outcomes. This study was designed as an exploratory, real-world pre–post intervention without a control group. As such, the findings should be interpreted as associations rather than causal effects.

### 2.3. Instruments

To identify ergonomic risk factors within the operating room environment, formal observational audits were conducted from 15 to 19 January 2024. Two trained observers independently evaluated 20 operating room nurses while they performed routine clinical duties, including instrument tray transport, equipment maneuvering, and radiation protection activities. The observed nurses were selected from the original cohort of 33 participants and were limited to day-shift staff because the highest-risk orthopedic procedures and manual handling activities predominantly occur during daytime operating hours.

Quantitative assessments were performed using the Operating Room Nursing Musculoskeletal Injury Prevention Awareness Questionnaire and the Ergonomic Action Checklist to evaluate intervention effectiveness.

A preliminary needs assessment was conducted to identify routine workflow challenges, focusing on high-risk tasks, equipment handling, and the physical demands associated with protective lead aprons. The same 20 nurses participated in one-on-one interviews conducted by a sports medicine physician to identify workplace ergonomic hazards, operational challenges, and perceived barriers to safe practice. The interview findings were analyzed descriptively and used to supplement the quantitative results by providing additional context regarding ergonomic challenges and staff perceptions of the workplace environment. This baseline audit recorded a total of 2227 deviations, averaging 111.4 deviations per nurse. Statistical analyses were performed using IBM SPSS statistics for Windows, Version 23.0 (IBM Corp., Armonk, NY, USA).

### 2.4. Content Validity and Reliability Assessment

The Ergonomic Action Checklist was adapted from the OSHA Musculoskeletal Disorder Checklist to evaluate 11 specific non-ergonomic actions commonly observed among operating room nurses. Content validity was assessed by a panel of five experts, including three sports medicine physicians and two nursing experts, resulting in an excellent Content Validity Index (CVI) of 1.00. The musculoskeletal injury prevention awareness questionnaire was developed based on relevant literature and clinical ergonomic principles. Content validity was evaluated by the same expert panel, yielding a Content Validity Index (CVI) of 0.88. The questionnaire also demonstrated acceptable psychometric properties with respect to internal consistency.

Reliability was assessed using Cronbach’s alpha coefficient, yielding a value of 0.80 during pilot testing (n = 5) and 0.92 in the formal study cohort, indicating good to excellent internal consistency among questionnaire items and supporting the reliability of the instrument for assessing musculoskeletal injury prevention awareness among operating room nurses.

To ensure consistency and objectivity in the observational audits, two designated observers underwent standardized training using clinical photographs and videos to establish uniform criteria for identifying non-ergonomic actions. Inter-rater reliability was evaluated in a pilot study in which the observers independently assessed 10 operating room nurses during routine clinical practice and recorded the occurrence of the 11 predefined ergonomic risk behaviors. Reliability was assessed using the Intraclass Correlation Coefficient (ICC) based on a two-way random-effects model with absolute agreement [ICC(2, 1)]. The checklist demonstrated excellent inter-rater reliability, with an overall ICC of 0.992 (95% CI, 0.985–0.996; *p* < 0.001), indicating consistency between observers and supporting the objectivity and reproducibility of the assessment tool.

### 2.5. Interventions

The primary risk factors for musculoskeletal injuries include unfavorable postures, excessive force exertion, repetitive movements, and prolonged physical stress. To address these issues, Human Factors Engineering (HFE) principles were applied to optimize the interaction between personnel, tools, and the work environment. Through systematic analysis, HFE identifies ergonomic deficiencies and guides improvements designed to mitigate workplace discomfort and fatigue.

Diagnostic Phase: The Standardized Nordic Musculoskeletal Questionnaire (NMQ) was utilized as a screening tool to assess symptom prevalence and identify potential hazards. The NMQ facilitates the early detection of at-risk workers, enabling subsequent, detailed hazard assessments using specific HFE methodologies [9].

Following the initial screening, the OSHA Musculoskeletal Disorder (MSD) Checklist was applied. This tool is particularly effective for high-intensity work environments involving prolonged manual handling. It prioritizes the identification of significant risk factors, estimates MSD probability, and informs the development of preventive and corrective strategies [10].

Intervention Strategies: Based on the identified etiology and risk factors, the intervention program was structured according to the Health Belief Model (HBM) to systematically address cognitive perceptions and physical barriers to behavior change. A multifaceted intervention program was developed comprising the following strategies:

1. Educational Interventions:
  - Development of a cross-disciplinary in-service training curriculum.
  - Creation and display of educational posters regarding MSD prevention.
2. Engineering and Environmental Interventions:
  - Redesign of surgical instrument trays to optimize weight and content.
  - Procurement of lightweight lead aprons.
  - Optimization of instrument placement and development of an ergonomic layout diagram.
  - Development of a specific human factors hazard assessment checklist.

### 2.6. Outcome Measures

Musculoskeletal injury prevention awareness was assessed using a 10-item, researcher-developed questionnaire with dichotomous (correct/incorrect) response options. The instrument evaluated nurses’ knowledge and awareness regarding common musculoskeletal injury sites, risk assessment methods, non-ergonomic working postures, preventive strategies, and the management of musculoskeletal discomfort.

Outcomes were evaluated across three primary domains: musculoskeletal injury prevention awareness, ergonomic compliance, and work-related musculoskeletal health outcomes.

1. Prevention Awareness: Changes in awareness were assessed using the Operating Room Nursing Musculoskeletal Injury Prevention Awareness Questionnaire. This instrument was administered at baseline and immediately post-intervention, with the post-intervention assessment conducted on 16 June 2024, to evaluate the efficacy of the ergonomic educational component.

2. Ergonomic Compliance: Behavioral adherence was measured using the Operating Room Nursing Staff Ergonomic Action Checklist. This tool documented the frequency of ergonomic deviations during critical tasks, such as carrying instrument trays, maneuvering equipment, and wearing lead aprons. Audits were conducted at baseline, immediately post-intervention, and at three- and six-month follow-up intervals.

3. Musculoskeletal Health Outcomes: Clinical impact was evaluated by monitoring self-reported symptoms, sick leave rates, and functional recovery. Data were aggregated from staff self-reports and unit administrative records from July to December 2024 to assess post-intervention effectiveness.

Collectively, these multidimensional metrics provided a comprehensive evaluation of both the immediate impact and mid-term sustainability of the ergonomic and educational intervention. The initial assessment and interviews span from 21 January to 25 January 2024, while the post-intervention observational period spanned from 25 July to 30 August 2024, with a total intervention interval of seven months.

## 3. Results

### 3.1. Needs Assessment of Ergonomic Hazards

The structured interviews with nursing staff identified specific high-risk activities and environmental hazards within the OR setting. Three primary categories of ergonomic risks were observed:

#### 3.1.1. Heavy Manual Lifting

Nurses consistently reported the manual handling of instrument trays and sterile wraps weighing ≥10 kg. The cumulative daily load per nurse was estimated at approximately 500 kg, with upwards of five items per surgery to and from the OR, for four to five surgeries a day.

#### 3.1.2. Equipment Maneuvering

Specialized operating tables (such as fluoroscopic tables for spinal surgery and fracture tables) frequently exceed 200 kg and often require manual positioning by a single nurse. The physical strain is exacerbated by long transport distances and narrow corridors within the facility.

#### 3.1.3. Radiation Protection Burden

Procedures necessitating mobile fluoroscopic imaging (e.g., fracture reduction surgery, percutaneous vertebroplasty) impose significant physical demands. Scrub nurses perform repetitive instrument transfers while wearing 6 kg lead aprons, whereas circulating nurses must maneuver mobile X-ray fluoroscopic units weighing up to 400 kg under similar lead protection. These combined factors significantly heighten the risk of injury to the shoulders, neck, lumbar spine, and knees.

### 3.2. Musculoskeletal Injury Prevention Awareness

**Figure 1:**
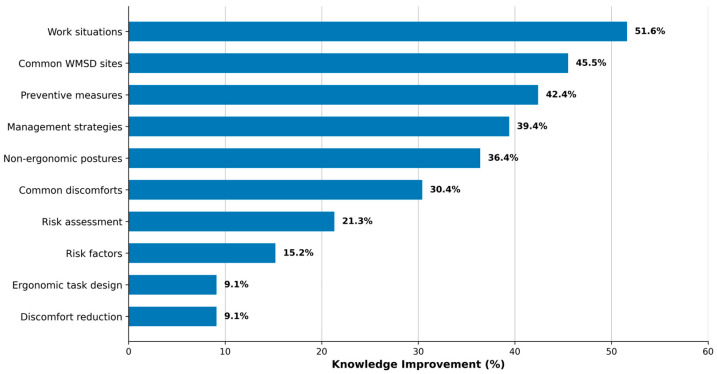
Item-level improvement in musculoskeletal injury prevention awareness among operating room nurses. Note: Percentage improvement in correct responses for each item of the Operating Room Nursing Musculoskeletal Injury Prevention Awareness Questionnaire following the multifaceted ergonomic intervention. Items are ranked from highest to lowest improvement. WMSD: work-related musculoskeletal disorder.

The Operating Room Nursing Musculoskeletal Injury Prevention Awareness Questionnaire (Supplementary File S1) was re-administered to the original cohort of 33 nursing staff. Post-intervention analysis revealed a substantial increase in awareness scores, rising from 68.7% at baseline to 98.8% (Table 2). Item-level improvements across the ten questionnaire domains, ranked in descending order, are presented in Figure 1, using the Operating Room Nursing Musculoskeletal Injury Prevention Awareness Questionnaire.

Overall, gains were more pronounced in domains related to recognizing high-risk work situations and preventive strategies, whereas improvements in ergonomic task design and management of discomfort were comparatively smaller.

### 3.3. Ergonomic Action Compliance

**Figure 2:**
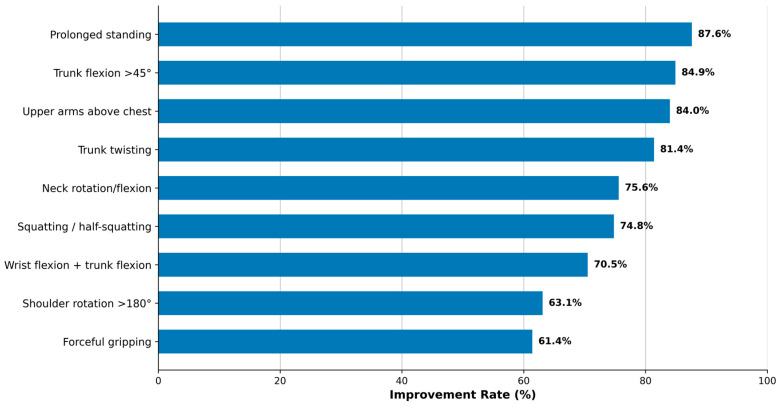
Improvement rates of non-ergonomic actions observed during operating room activities following the intervention. Note: This figure shows the percentage reduction in observed non-ergonomic postures and actions, as assessed using the Ergonomic Action Checklist before and after the multifaceted ergonomic intervention. Items are ranked from highest to lowest improvement. The greatest improvements were observed in trunk-related postures and prolonged standing, whereas improvements in upper extremity postures were comparatively smaller.

Field observations of 20 nurses were conducted between 25 July and 30 August 2024, focusing on high-risk tasks such as carrying instrument trays, maneuvering equipment, and wearing lead aprons. Audits using the Ergonomic Action Checklist revealed a marked decline in ergonomic deviations. The total number of unsafe actions dropped from 2227 at baseline to 440 post-intervention, corresponding to a reduction in the mean number of deviations per nurse from 111.3 to 22.0 (Table 3). Improvement rates of individual non-ergonomic actions are illustrated in Figure 2. Reductions were most pronounced in prolonged standing and trunk-related postures, while improvements in upper extremity postures were comparatively smaller. Follow-up audits at three- and six-months post-intervention demonstrated a sustained downward trend, with mean deviations further decreasing to 21.2 and 11.6, respectively. These findings indicate that the behavioral improvements were sustained for at least six months.

### 3.4. Work-Related Musculoskeletal Outcomes

Analysis of sick leave records from July to December 2024 indicated that only one staff member required leave attributed to a work-related musculoskeletal injury. In contrast to the previous year, during which three of 33 staff members (9.1%) in the same cohort required sick leave due to work-related musculoskeletal discomfort, only one of 33 staff members (3.0%) required sick leave during the post-intervention period, representing a notable decrease. Staff members with a history of shoulder, neck, or lower back discomfort reported symptom alleviation following the intervention and rehabilitation. Additionally, qualitative feedback suggested that occupational musculoskeletal discomfort became more manageable with appropriate rest. Qualitative feedback was evaluated anonymously by three sports medicine physicians in a non-clinical setting.

## 4. Discussion

This study provides evidence that a structured intervention, integrating ergonomic education with human factors engineering, has been observed to improve ergonomic awareness, reduced non-ergonomic behaviors, and mitigated musculoskeletal symptoms among operating room (OR) nursing staff. The heterogeneous patterns of improvement observed across awareness domains and ergonomic actions (Figure 1 and Figure 2) highlight the complementary roles of educational and engineering components within the intervention. Specifically, substantial improvements in awareness and reductions in non-ergonomic behaviors were observed following the intervention and were sustained at three- and six-month follow-ups. These findings underscore the critical value of combining educational initiatives with structural modifications in high-risk clinical environments.

The high baseline prevalence of musculoskeletal symptoms in this cohort mirrors global evidence, which indicates that 60–80% of nurses suffer from work-related musculoskeletal disorders (WMSDs), predominantly affecting the neck, shoulders, and lower back [1,5,11]. Data from Taiwan similarly corroborate this burden, linking symptoms to physically demanding tasks such as heavy lifting, prolonged standing, and repetitive overhead reaching [3]. This aligns with early ergonomic evaluations by Garb and Dockery, who identified the maneuvering of heavy carts and equipment as critical risk factors for back injuries in the perioperative setting [12]. A 2023 meta-analysis confirms that biomechanical overload remains a primary driver of WMSDs, with a pooled prevalence exceeding 70% among nurses worldwide [1]. These data correspond to the inherent physical rigors of OR nursing observed in this study, where staff routinely manipulate ≥200-kg operating tables, carry heavy instrument trays, and wear lead aprons while maintaining unfavorable postures in constrained spaces.

The efficacy of this intervention can be attributed to a synergistic effect of behavioral and environmental modifications. While previous studies have examined either behavioral determinants or ergonomic interventions in isolation, this study suggests the effectiveness of an integrated, theoretically grounded, and multi-component ergonomic prevention strategy in a real-world OR setting. This approach uniquely integrates behavioral theory-driven risk identification with human factors-based system redesign, bridging the gap between epidemiological findings and practical real-world workplace intervention. The educational component likely enhanced knowledge, risk perception, and self-efficacy. These factors are recognized as key drivers of behavior change within the Health Belief Model (HBM). In the context of the HBM, this targeted education has shown evidence of heightened perceived susceptibility and perceived severity regarding work-related injuries. By internalizing these risks, the nursing staff developed a stronger perceived threat, which served as the primary cognitive catalyst for behavioral change. This theoretical basis is substantiated by recent findings from Attia et al., who demonstrated a significant correlation between ergonomic awareness and musculoskeletal disorders, thereby confirming that enhancing awareness is a fundamental prerequisite for prevention [13]. Although prior studies suggest that education can improve posture and reduce symptoms [14,15], contemporary research increasingly argues that education alone is insufficient for long-term impact. A 2024 expert review emphasized a paradigm shift toward multi-component ergonomic prevention strategy, advocating for workplace redesign to eliminate biomechanical hazards at the source [16]. Consistent with this view, recent systematic reviews indicate that engineering controls (e.g., equipment optimization and load reduction) outperform isolated training programs in healthcare settings [17,18]. Crucially, while education addresses the psychological intent to change, the HFE-based structural modifications implemented in this study were essential for overcoming perceived barriers. By physically reducing the weight of instrument trays and the burden of lead aprons, the intervention ensured that ergonomic compliance was no longer perceived as an added difficulty but as a feasible workplace reality.

This study corroborates these contemporary findings by implementing specific human factors engineering strategies, including tray weight reduction, workspace reorganization, and the adoption of lighter lead aprons. These modifications directly reduce biomechanical load, thereby minimizing reliance on individual vigilance and promoting sustained adherence to safe movement patterns. This systems-oriented approach aligns with sociotechnical safety principles, which posit that durable occupational health improvements stem from modifying the work system rather than solely focusing on worker behavior [19]. The sustained adherence observed at the six-month follow-up is consistent with the theoretical role of self-efficacy in promoting health-related behavioral change. In our baseline survey of the same cohort, self-efficacy was identified as the strongest predictor of preventive ergonomic behaviors (OR = 13.508, *p* < 0.001). However, post-intervention self-efficacy was not assessed; therefore, the extent to which changes in self-efficacy contributed to the observed outcomes remains uncertain. Given the relatively small sample size, this large odds ratio should be interpreted cautiously, as effect estimates from this exploratory logistic regression analysis may be unstable and subject to overestimation. Future studies incorporating repeated assessments of self-efficacy will be important to better understand the relationship between behavioral change and sustained ergonomic practice.

It is possible that more experienced nurses, while possessing established workflow habits, may demonstrate varying degrees of adaptability to ergonomic interventions. Conversely, increased clinical experience may also enhance awareness of occupational risk, potentially facilitating behavioral change. Although this study did not formally stratify outcomes based on years of experience or credentials, the strong association observed with self-efficacy suggests that individual behavioral factors may play a critical role in the adoption of ergonomic practices. Future studies should further explore this relationship.

As nurses successfully applied ergonomic skills within an optimized environment, their confidence in managing occupational risks grew, reinforcing a virtuous cycle of behavioral maintenance that is central to the HBM framework. The sustained decline in non-ergonomic actions and the reduction in sick leave and symptoms highlight the clinical relevance of this dual approach. Furthermore, evidence suggests that such multifaceted interventions may not only reduce pain [20] and sickness absence [21] but also enhance well-being and workforce retention [22]. While a reduction in sick leave days was observed following the intervention, this outcome may be influenced by multiple external factors not controlled for in the study design. These may include institutional policy changes, staffing adjustments, seasonal variations, and broader healthcare system influences, particularly in the post-pandemic period [23]. The observed reduction in musculoskeletal-related sick leave should be interpreted cautiously.

Thus, integrating ergonomic training with environmental redesign appears essential for sustaining a resilient OR nursing workforce. Although the current study specifically targeted OR nurses, the core components of this intervention, namely ergonomic education and environmental modification, are highly transferable to other clinical settings. High-intensity units, such as intensive care units (ICUs) and emergency departments, share similar physical demands, including patient handling and prolonged static postures [24]. Therefore, adapting this multifaceted protocol to the specific workflows of other departments could yield comparable benefits in mitigating musculoskeletal disorders across the healthcare spectrum [25].

Several limitations warrant consideration. First, the single-center design and modest sample size may limit generalizability and statistical robustness. Although statistically significant associations were observed for certain variables, these findings should be interpreted with caution, as the limited sample size may increase the risk of overestimation and reduce the stability of the estimates. While this constraint may affect formal statistical power, the consistency of the observed improvements suggests a meaningful trend toward enhanced ergonomic compliance.

Second, the absence of a control group precludes causal inference. The observed improvements may be influenced by alternative explanations, including increased awareness due to observation (Hawthorne effect), temporal trends, or other external factors. Although improvements were sustained over a six-month period, the study design does not allow attribution of these changes solely to the intervention.

Third, reliance on direct observation and partially validated measurement tools may introduce bias and variability. The magnitude of observed improvements may partly reflect observer influence or short-term behavioral adaptation rather than sustained long-term change. Expert-validated tools were used, with acceptable content validity (CVI = 0.88), inter-rater reliability of the Ergonomic Action Checklist was evaluated in a pilot assessment involving two trained raters observing 10 operating room nurses. Excellent agreement was demonstrated (ICC = 0.992, 95% CI 0.985–0.996). Standardized protocols and trained observers likely mitigated variability; however, these factors may still limit measurement precision. Thus, reliability testing was only performed in a limited sample and further validation in larger cohorts remains warranted. Furthermore, the awareness questionnaire has not undergone full psychometric validation, which stands to be a limitation of the study.

A notable limitation of our study is the multifaceted design of the intervention. The program combined educational modules, ergonomic equipment modifications, workflow adjustments, and continuous audit feedback—implemented simultaneously. While this integrative approach reflects real-world practice and likely contributed to the overall success, it inherently prevents us from isolating the effect of any single component. As a result, we cannot determine which specific element had the greatest impact. Future research employing controlled or factorial designs, where individual components are introduced stepwise or independently, will be needed to pinpoint the specific drivers of change and optimize future interventions.

Finally, post-intervention self-efficacy was not reassessed, which limits our ability to determine whether changes in self-efficacy contributed to the observed behavioral improvements.

Future studies should incorporate multi-center designs with control groups to enhance generalizability and causal inference. Additionally, the use of objective assessment tools, such as wearable motion sensors or AI-based posture detection, is recommended to improve measurement accuracy. Longer-term follow-up beyond 12 months, as well as formal reliability testing and stratification of qualitative symptoms in a clinical setting, are warranted to further strengthen methodological rigor and evaluate the durability of intervention effects.

This intervention was implemented within an institutional occupational health framework. As part of ongoing safety initiatives, the hospital supported ergonomic modifications, including lighter lead aprons and reduced instrument tray weights. These changes aligned with broader efforts to reduce occupational hazards and demonstrate real-world feasibility within existing programs. However, cost-effectiveness was not formally evaluated and warrants further study.

## 5. Conclusions

This study suggests that integrating ergonomic education with human factors engineering strategies is associated with a reduction in musculoskeletal symptoms. Previous studies have demonstrated that both single-component ergonomic interventions and multifaceted programs can improve musculoskeletal health among nursing staff [16,17,18,20]. Building upon this evidence, our study specifically focused on orthopedic operating room nurses, a highly specialized workforce exposed to unique ergonomic hazards including repetitive heavy manual handling, prolonged use of lead aprons, and manipulation of large surgical equipment. Rather than evaluating isolated intervention components, our program deliberately integrated the Health Belief Model (HBM) with Human Factors Engineering (HFE) into a multifaceted intervention incorporating behavioral education, system-level ergonomic modifications, direct observational ergonomic audits, and longitudinal follow-up. By simultaneously addressing behavioral and environmental determinants, this approach may enhance ergonomic awareness, improve compliance with safe work practices, and support sustained behavioral change. The observed improvements likely reflect the combined effects of behavioral and environmental modifications rather than any single intervention component.

These findings support a growing emphasis in occupational health on integrating behavioral and system-level strategies to address work-related musculoskeletal disorders. As an exploratory real-world quality improvement study, our findings suggest that integrating the Health Belief Model with Human Factors Engineering is a feasible and practical approach for promoting safer workplace behaviors within specialized operating room environments. However, the observed associations should be interpreted cautiously given the single-center pre-post design, relatively small sample size, and absence of a control group, which limit causal inference and generalizability. Nevertheless, this study provides a practical framework for implementing multifaceted interventions in high-risk surgical settings and generates hypotheses for future investigation. Further multicenter controlled studies are warranted to confirm these findings, determine their generalizability, evaluate cost-effectiveness, identify the individual contributions of specific intervention components, and explore the effectiveness of this multifaceted intervention across diverse surgical specialties and other high-risk healthcare environments.

## Figures and Tables

**Table 1:** Logistic Regression Analysis of Musculoskeletal Injury Prevention Behaviors.

Predictors	Odds Ratio	95% CI	*p*-Value
Nursing credentials	2.126	1.211–3.730	0.009 **
Musculoskeletal Injury (MSI)	0.986	0.574–1.696	0.960
Marital status	0.993	0.597–1.650	0.987
Musculoskeletal symptoms	0.866	0.524–1.430	0.573
Occupation	0.903	0.532–1.534	0.706
Work load	1.316	0.793–2.183	0.288
Gender and Disease	1.114	0.679–1.828	0.670
Perceived Threat	0.683	0.204–2.290	0.537
Perceived Severity	1.744	0.598–5.085	0.308
Perceived Benefits	1.163	0.336–4.023	0.812
Perceived Barrier	2.381	0.637–8.909	0.197
Cues to action	0.707	0.120–4.153	0.701
Self-efficacy	13.508	4.127–44.217	<0.001 *

Note: Odds ratios (ORs) and 95% confidence intervals (CIs) were estimated using multivariable logistic regression analysis to identify predictors of musculoskeletal injury prevention behaviors. Model fit was assessed using Cox and Snell R^2^ and Nagelkerke R^2^. The Hosmer–Lemeshow goodness-of-fit test indicated an adequate model fit (χ^2^(8) = 11.938, *p* = 0.154). (*p* < 0.05, * *p* < 0.01, ** *p* < 0.001).

**Table 2:** Comparison of Musculoskeletal Injury Prevention Awareness Among Operating Room Nurses Before and After the Intervention (N = 33).

Item	Pre-Intervention (%)	Post-Intervention (%)	Difference (%)
1. Common sites of work-related musculoskeletal disorders (WMSDs)	45.4	90.9	45.5 *
2. Risk assessment methods for musculoskeletal injuries	78.7	100.0	21.3 *
3. Common musculoskeletal discomforts encountered	69.6	100.0	30.4 *
4. Non-ergonomic postures during OR duties	63.6	100.0	36.4 *
5. Work situations prone to musculoskeletal discomfort	48.4	100.0	51.6 *
6. Risk factors for musculoskeletal discomfort	84.8	100.0	15.2 *
7. Ergonomic task design to reduce musculoskeletal discomfort	90.9	100.0	9.1 *
8. Methods to reduce occurrence of musculoskeletal discomfort during routine duty	90.9	100.0	9.1 *
9. Preventive measures for musculoskeletal injuries during clinical practice	54.5	96.9	42.4 *
10. Management strategies for musculoskeletal discomfort	60.6	100.0	39.4 *
Overall mean	68.7	98.8	30.1 *

Overall awareness scores improved significantly from 68.7 ± 11.2 to 98.9 ± 2.8 following the intervention (mean difference 30.2 ± 10.1, 95% CI 26.6–33.8; * paired *t*-test *p* < 0.001).

**Table 3:** Comparison of Non-Ergonomic Actions Before and After the Intervention (N = 20).

Hazardous Factor (Ergonomic Risk)	Pre-Intervention (Mean ± SD)	Post-Intervention (Mean ± SD)	Paired *t*-Test (*p*-Value)
Total Frequency of Actions	111.3 ± 4.8	22.0 ± 3.0	<0.001 *
Neck: rotation or forward bending > 20°	10.1 ± 1.8	2.5 ± 0.9	<0.001 *
Shoulders: upper arms raised > chest level	17.5 ± 1.6	2.8 ± 1.2	<0.001 *
Trunk: forward flexion > 45°	19.3 ± 2.5	2.9 ± 1.4	<0.001 *
Trunk: twisting	14.8 ± 1.9	2.8 ± 1.2	<0.001 *
Prolonged standing without back support	25.0 ± 2.3	3.1 ± 1.5	<0.001 *
Squatting or half-squatting posture	7.2 ± 1.3	1.8 ± 0.8	<0.001 *

* Paired-sample *t*-test analysis demonstrated a significant reduction in the frequency of all non-ergonomic actions (*p* < 0.001).

## Data Availability

The original contributions presented in this study are included in the article/Supplementary Materials. This study was conducted as part of an institutional quality improvement program, and the underlying raw data are owned by the institution’s Quality and Care Department. Therefore, the raw dataset is not publicly available. Upon reasonable request to the corresponding author, institutional permission can be sought to share de-identified data in accordance with institutional policies.

## Supplementary Materials

The following supporting information can be downloaded at: https://www.mdpi.com/article/10.3390/healthcare14142046/s1, Supplementary File S1: Musculoskeletal Discomfort Questionnaire.

## Abbreviations

The following abbreviations are used in this manuscript:

OR	Operating room
WMSDs	Work-related musculoskeletal disorders
HFE	Human Factors Engineering
MSI	Musculoskeletal Injury
ORs	Odds ratios
CIs	Confidence intervals
NMQ	Nordic Musculoskeletal Questionnaire
MSD	Musculoskeletal Disorder
OSHA	Occupational Safety and Health Administration
HBM	Health Belief Model
ICUs	Intensive care units
