# Supplementary material for: Integrating Health Belief Model and Human Factors Engineering to Prevent Musculoskeletal Injuries Among Operating Room Nurses: A Quality Improvement Prospective Pilot Study
Source: Healthcare (Basel). 2026 Jul 8;14(14):2046. doi: 10.3390/healthcare14142046 (PMC13411017; doi:10.3390/healthcare14142046)
Supplement: Supplementary file 1 [file healthcare-14-02046-s001.zip › healthcare-4352633-supplementary.pdf]

Dear Colleague,

Thank you for participating in this survey. The purpose of this survey is to understand the musculoskeletal injuries experienced by operating room nurses. We hope that the results of this study can serve as a reference for improving the effectiveness of musculoskeletal injury prevention among operating room nurses, thereby enhancing their health. The questionnaire is divided into three parts, with explanations provided before each section. Your responses will be anonymous, and all information provided will be kept strictly confidential. Please answer according to your true feelings. Thank you again for your cooperation!

Best regards,

嘉南藥理大學醫務管理系
指導教授：陳俞成 副教授
研 究 生：李佩珊

Musculoskeletal Discomfort Questionnaire

Part 1: Personal Information

Please. mak '✓' in the ☐ that coespons to you conition (single choice questions)

1. Gender: ☐Male ☐Female
2. Age: ☐Below 30 years old ☐31-40 years old ☐Above 41 years old
3. Marital Status: ☐Married ☐Single ☐Divorced
4. Have Children: ☐Yes ☐No
5. Education Level: ☐College ☐University or above
6. Education Level: ☐College ☐University or above
7. body weight:______ kilograms Height:________ Centimeters (If BMI is unknown, please provide height and weight).
8. BMI ☐<18.5 ☐≤18.5-24 ☐>24.1
9. Job Grade: ☐N ☐N1 ☐N2 ☐N3 ☐N4 or above
10. Years of Nursing Experience: ☐<7 years ☐8-14 years ☐14 years or more
11. Years of Operating Room Experience: ☐<7 years ☐8-14 years ☐14 years or more
12. Average Weekly Working Hours: ☐ ≤48 hours☐> 48 hours
13. Shift Work: ☐Yes ☐No
14. Department: ☐General Surgery ☐Obstetrics and Gynecology ☐Orthopedic Trauma ☐Plastic Surgery ☐Proctology ☐Ophthalmology ☐Specialist Nurse
15. Daily Standing Time: ☐≤6 hours ☐>6 hours

Part 2: Musculoskeletal Injury Severity Assessment Form

Please mark '✓' in the ☐ that coresponds to your condition

1. Have you experienced any musculoskeletal injury symptoms in the past year? ☐ Yes ☐ No

2. Location(s) of your musculoskeletal injury: ☐ No injury occurred ☐ One injured area ☐ Two or more injured areas
3. In the past year, where have you experienced musculoskeletal discomfort? ☐ No discomfort ☐ Neck ☐ Left shoulder ☐ Right shoulder ☐ Upper back ☐ Lower back / lumbar area ☐ Thigh ☐ Buttocks ☐ Left elbow ☐ Right elbow ☐ Left wrist ☐ Right wrist ☐ Left knee ☐ Right knee ☐ Left calf ☐ Right calf ☐ Left ankle ☐ Right ankle (multiple selections possible)
4. Primary symptoms: ☐ No symptoms ☐ Soreness ☐ Pain ☐ Numbness ☐ Difficulty moving ☐ Muscle weakness (multiple selections possible)
5. Frequency of symptoms: ☐ No symptoms ☐ Almost every day ☐ About once a week ☐ About once a month ☐ About once every six months ☐ Once a year or less
6. How long have you experienced soreness, discomfort, or joint mobility issues in your body parts?
☐ No symptoms ☐ Less than 1 month ☐ 1-3 months ☐ 3-6 months ☐ 6 months to 1 year
7. How has the symptom affected you? ☐ No impact at all ☐ Slight reduction in work capacity ☐ Significant decrease in work capacity ☐ Slight impact on daily life ☐ Severe impact on daily life ☐ Unable to function at all

Part 3: Musculoskeletal Health Beliefs Scale

1. I believe that I may develop musculoskeletal injuries in the future, indicated by soreness, pain, numbness, difficulty moving, and muscle weakness in various parts of my body.<br><br>Explanation: The options represent the following meanings:<br>**5: Very likely 4: Likely 3: Uncertain 2: Unlikely 1: Very unlikely**	
1. There may be musculoskeletal injuries in the next three months.	5 4 3 2 1
2. There may be musculoskeletal injuries in the next six months.	5 4 3 2 1
2. I believe that if I experience musculoskeletal injuries in the future, the severity of their impact on my work and life could be:<br><br>Explanation: The options represent the following meanings:<br>**5: Very likely 4: Likely 3: Uncertain 2: Unlikely 1: Very unlikely**	
1. The impact of musculoskeletal injuries on rest	5 4 3 2 1
2. The impact of musculoskeletal injuries on sleep	5 4 3 2 1
3. The impact of musculoskeletal injuries on leisure physical activities	5 4 3 2 1
4. The impact of musculoskeletal injuries on daily life routines and activities	5 4 3 2 1
5. The impact of musculoskeletal injuries on work efficiency	5 4 3 2 1
6. The impact of musculoskeletal injuries on work emotions.	5 4 3 2 1
7. The impact of musculoskeletal injuries on coworker relationships	5 4 3 2 1
8. The impact of musculoskeletal injuries on nurse-patient relationships	5 4 3 2 1
9. The impact of musculoskeletal injuries on overall physical health	5 4 3 2 1
10. The impact of musculoskeletal injuries on overall mental health	5 4 3 2 1
11. The impact of musculoskeletal injuries on medical expenses	5 4 3 2 1
3. I believe that adopting behaviors to prevent musculoskeletal injuries could bring the following benefits:<br><br>Explanation: The options represent the following meanings:<br>**5: Very likely 4: Likely 3: Uncertain 2: Unlikely 1: Very unlikely**	
1. Avoid pain and discomfort	5 4 3 2 1

2.Promote relaxation	5	4	3	2	1
3.Improve sleep quality	5	4	3	2	1
4.Enhance work efficiency	5	4	3	2	1
5.Improve work mood	5	4	3	2	1
6.Better interactions and interpersonal relationships	5	4	3	2	1
7.Avoid job loss	5	4	3	2	1
8.Enhance physical strength and reduce the likelihood of illness	5	4	3	2	1
9.Reduce medical expenses	5	4	3	2	1
10.Decrease the number of sick days	5	4	3	2	1
4. I believe the difficulties in adopting behaviors to prevent musculoskeletal injuries at work are...<br><br>Explanation: The options represent the following meanings:<br>**5: Very likely 4: Likely 3: Uncertain 2: Unlikely 1: Very unlikely**					
1.Implementing preventive measures for musculoskeletal injuries is very troublesome	5	4	3	2	1
2.Correct work postures are impractical	5	4	3	2	1
3.Heavy workload leaves no time for prevention	5	4	3	2	1
4.Lack of understanding of the causes of musculoskeletal injuries	5	4	3	2	1
5.Unclear on how to prevent musculoskeletal injuries	5	4	3	2	1
6.Affect nursing work efficiency	5	4	3	2	1
7.Insufficient nursing skills lead to incorrect postures	5	4	3	2	1
8.Easily stressed during work, neglecting proper posture	5	4	3	2	1
9.Do not believe in the effectiveness of preventive measures	5	4	3	2	1
10 Poor design of hospital hardware facilities	5	4	3	2	1
5. During your studies or work, have you had any of the following learning experiences related to musculoskeletal injuries:<br><br>Explanation: The numbers in the options represent the significance of the data.<br>**If yes, circle "Yes". If no, circle "No".**					
1.Have you ever participated in "Prevention of Musculoskeletal Injuries" related educational training organized by your workplace hospital?	Yes	No			
2.Have you ever read brochures, manuals, or articles in newspapers and magazines about musculoskeletal injuries?	Yes	No			
3.Have you ever watched (listened to) TV or radio content related to musculoskeletal injuries?	Yes	No			
4.Have you ever seen information related to musculoskeletal injuries on the internet?	Yes	No			
5.Information on Musculoskeletal Injuries from Friends and Family	Yes	No			
6.I have personal experience with seeking medical treatment for musculoskeletal injuries.	Yes	No			
7.I was reminded by a colleague to pay attention to my work posture.	Yes	No			
8.The school I attended once provided educational training on 'preventing musculoskeletal injuries	Yes	No			
6. I can practically implement and achieve the following behaviors or postures in my routine nursing work:<br><br>Explanation: The numbers in the options represent the following meanings:<br>**5: Always 4: Frequently 3: Sometimes 2: Rarely 1: Never**					
1.I adjust the work surface (standing desk)) to a height that is suitable for me while working.	5	4	3	2	1

2.When working for long periods of standing or sitting, I make sure to frequently change positions.	5	4	3	2	1
3.When performing nursing tasks, I am able to keep my upper body straight without bending over.	5	4	3	2	1
4.When performing nursing tasks, I am able to avoid twisting my body.	5	4	3	2	1
5.When handling objects at work, I squat down to lift them?	5	4	3	2	1
6.When feeling fatigued while performing nursing tasks, I take a break from work to rest.	5	4	3	2	1
7.I do not lift items weighing more than 10 kilograms during work.	5	4	3	2	1
8.When moving patients, I keep them close to my body.	5	4	3	2	1
9.When moving patients, I seek help from others (colleagues).	5	4	3	2	1
10.When lifting heavy objects, I keep the items close to my body.	5	4	3	2	1
11.When lifting heavy objects, I seek assistance from others (colleagues).	5	4	3	2	1
12.I wear a soft back support,corset and personal protective equipment while working.	5	4	3	2	1
13.I do stretching exercises before starting work	5	4	3	2	1
7. I can ensure prevention of musculoskeletal injuries for the following behaviors or postures:<br><br>Explanation of options:<br>**5: Very confident 4: Confident 3: Uncertain 2: Not confident 1: Not at all confident**					
1. Do you ensure that you can adjust your work desk (standing desk) to a height that suits you during work?	5	4	3	2	1
2. When needing to stand or sit for prolonged periods at work, do you ensure that you can frequently change positions?	5	4	3	2	1
3. When performing nursing duties, do you ensure that you can keep your upper body straight without bending over?	5	4	3	2	1
4. When performing nursing duties, do you ensure that you can avoid twisting your body?	5	4	3	2	1
5. When handling objects at work, do you ensure that you can squat down to lift them?	5	4	3	2	1
6. When feeling tired during nursing duties, do you ensure to take breaks and rest temporarily?	5	4	3	2	1
7. Do you ensure that you can lift objects weighing more than 10 kilograms during work?	5	4	3	2	1
8. Do you ensure that you can keep the patient close to your body when transferring them?	5	4	3	2	1
9. Do you ensure that you can seek help from others (colleagues) when transferring patients?	5	4	3	2	1
10. Do you ensure that you can keep the objects close to your body when lifting heavy items?	5	4	3	2	1
11. Do you ensure that you can seek help from others (colleagues) when lifting heavy objects?	5	4	3	2	1
12. Do you ensure to wear a soft back brace, lumbar support, and personal protective equipment while working?	5	4	3	2	1
13. Do you perform stretching exercises before work?	5	4	3	2	1

This concludes the questionnaire. Thank you for your responses!
